# EUS-Assisted Evaluation of Rectal Varices before Banding

**DOI:** 10.1155/2013/619187

**Published:** 2013-05-27

**Authors:** Malay Sharma, Praveer Rai, Raghav Bansal

**Affiliations:** ^1^Department of Gastroenterology, Jaswant Rai Speciality Hospital, Saket, Meerut, PIN-250 001 Uttar Pradesh, India; ^2^SGPGI, Lucknow, Uttar Pradesh, India; ^3^Gastroenterology Fellow, Mount Sinai Elmhurst Hospital Center, 80-15 41 Avenue Apt 741, Elmhurst, NY 11373, USA

## Abstract

Rectal varices are an important cause of bleed. The bleeding can be sometimes fatal. Endoscopic management is possible and is generally done in emergency situation. Rectal variceal banding is useful. Hemodynamic evaluation has shown that the blood flow in rectal varices is from above downwards; however, the site of banding of rectal varices is unclear. This case series shows that the rectal varices should be banded at the highest point of inflow.

## 1. Introduction

**Figure 1:**
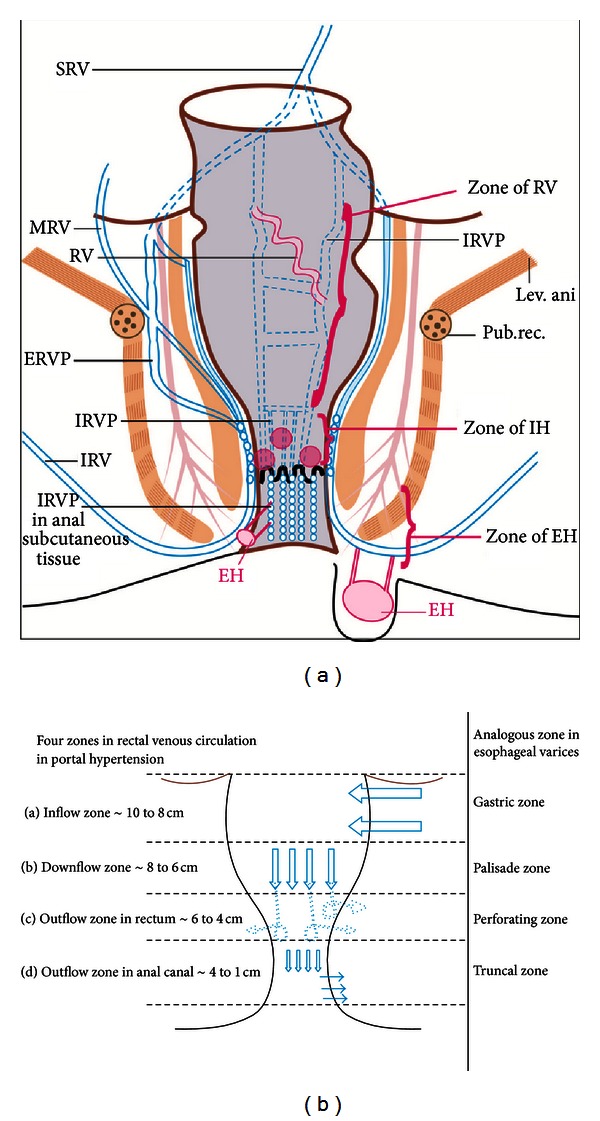
(a) The superior rectal vein (SRV) divides into two branches, which enter the lateral wall of rectum, about 10 cm above the dentate line. The middle and inferior rectal veins (MRV & IRV), empty into the caval system. The rectal veins form two plexuses, an internal one lying in the submucosa and the corresponding anal “Subcutaneous” tissue and an external one lying outside the muscular wall of the bowel below the level of the peritoneal reflection. The intrinsic rectal venous plexus consists of two groups of veins draining in opposite direction. The inferior group passes down to form the inferior rectal veins, and dilation of this group leads to formation of external hemorrhoids. The vessels of the superior group in the anal columns lead to the formation of internal hemorrhoids and in the rectum lead to the formation of rectal varices. (b) Four distinct zones of mucosal circulation are seen in rectum with similarity to esophageal circulation. The inflow area is analogous to the gastric zone, the downflow area is analogous to the palisade zone, the outflow area in rectum is analogous to the perforating zone, and the outflow area in anal canal is analogous to the truncal zone of esophageal varices.

Rectal varices (RVs) are an important cause of lower gastrointestinal bleed (LGIB) in portal hypertension (PHT) and have been reported to occur in 44% to 89% of cases of cirrhosis [1–3]. RVs are dilated submucosal portosystemic communications which extend from midrectum to the anorectal junction and are considered distinct from internal hemorrhoids, which are submucosal arteriovenous communications of the anorectal vascular plexus [4]. Pelvic angiography studies have revealed that most of the submucosal portosystemic communications (PSCs) of RVs have hepatofugal inflow to intrinsic rectal venous plexus (IRVP) through the wall of rectum by branches of superior rectal vein (SRV): a tributary of inferior mesenteric vein [5]. The SRV inflow to IRVP occurs at about 10 cm distance in lateral wall of rectum and the middle and inferior rectal veins (IRV) act as the outflowing channels [5] (Figures 1(a) and 1(b)). Four distinct zones of PSC have been shown in portal hypertension (PHT) near the esophagus, and anatomical studies have shown that similar portosystemic communications exist in rectum in PHT in relation to IRVP (Figure 1(b)) [6, 7]. 

The suspicion of RVs as the cause of bleeding can be made with a high index of suspicion when lower GI bleed is seen in absence of hemorrhoids, and colonoscopy shows blood in rectum. Bleeding usually happens from endoscopically evident rectal varices (EERV) but sometimes bleed can occur from varices, which are endoscopically inevident (EIERV). Endoscopic ultrasound (EUS) has been shown to be more sensitive in diagnosis of EIERV [8–10]. Endoscopic and EUS correlation of RVs has shown that RVs, classified as tortuous, nodular, and tumorous on endoscopic examination, have corresponding appearances on rectal EUS as single, multiple, and innumerable submucosal veins, respectively [11]. The hemodynamic evaluation (HDE) of RVs by EUS is routinely done at some centers to assess parameters like the site, size, velocity, or direction of flow [9, 12]. The HDE of these parameters can offer therapeutic advantage before the selection of endoscopic or interventional radiological therapy [13]. This case series was done to evaluate the role of EUS in detection of RVs and the role of HDE before selecting the optimal site of endotherapy.

## 2. Material and Method 

Between Jan 2009 and October 2011 sixteen consecutive patients with portal hypertension and LGIB underwent evaluation for rectal varices. Patient consent was obtained prior to the procedure. Ethics committee of the Institution approved the study. The diagnosis of RVs was made by endoscopic examination or EUS in five cases. Endoscopic examination included initial proctoscopic/sigmoidoscopic examinations followed by a complete colonoscopy to rule out any other cause of bleeding. Patient confirmed or suspected to have RVs on endoscopy underwent diagnostic and hemodynamic evaluation by a radial endoscopic ultrasound scope (EUS) in the same session. The radial probe was advanced to 20 cm distance in rectum, which was filled with 100 to 250 mL of water. A color Doppler box with a focal distance of 3 to 4 cm was applied for entire circumference (360 degree) around the probe and continuous color Doppler application was done during slow withdrawal to the anus. The HDE of the venous circulation was done from higher up in rectum up to the anal verge and included the evaluation of site size and number of RVs, pararectal varices, and perforators (inflowing or outflowing) at three distances in rectum: 8 to 10 cm, 6 to 8 cm, and 4 to 6 cm. HDE was continued in the anal canal and the upper anal canal was identified by the puborectalis sling on EUS.

RVs were identified in the submucosal layer of rectal wall. The pararectal varices were identified in a location outside the wall of rectum. The perforators were identified as the communication traversing through the muscularis propria of rectal wall. The inflowing perforators were identified as flow signals towards the probe (red color) and outflowing perforators were identified as flow signals away from the probe (blue color). After HDE variceal ligation of RVs was done. If RVs were not suitably evident on endoscopy for banding, the information available on EUS was used for selection of site of banding.

## 3. Result and Discussion

**Figure 2:**
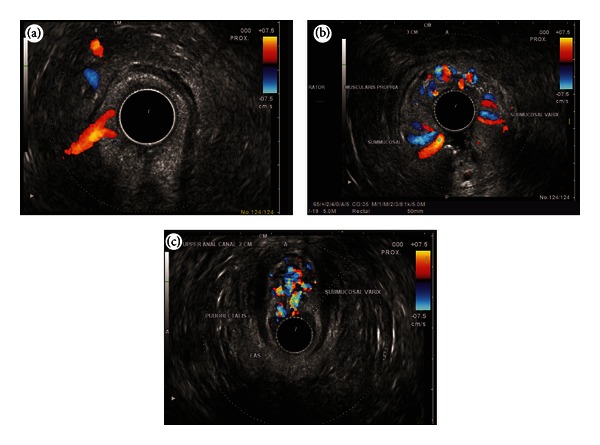
(a) An inflowing perforator of 3 mm diameter noted in the right lateral wall of rectum. (b) As the scope is pulled down towards the anorectal junction, the varices are seen circumferentially in the submucosa. (c) The varices are seen going anteriorly towards the genital plexus.

**Figure 3:**
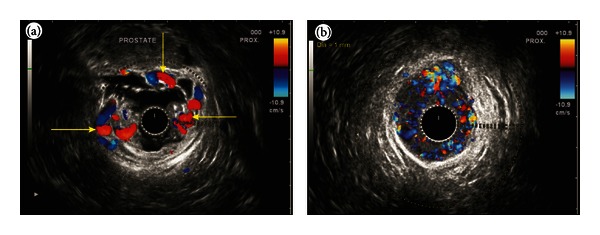
(a) As the scope is withdrawn towards the lower rectum, the submucosal varices were seen in anterior and lateral wall of rectum. (b) As the scope is pulled through the anus multiple small perforators <1 mm diameter were seen going through the muscular layer of anal canal.

**Figure 4:**
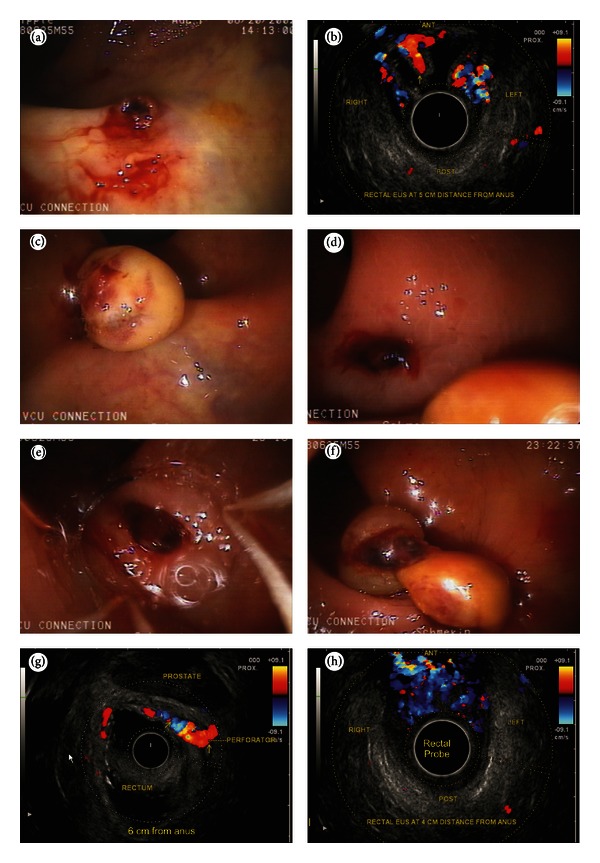
(a) An ulcer covered by a clot gives appearance of Dieaulafoy's ulcer. Clot could not be removed by flushing. (b) At about 6 cm distance in rectum inflowing perforators were noted in the submucosa of rectum. No pararectal varices were seen. (c) A band is applied on the clot as rectal varices were demonstrated by EUS under the ulcer. (d) The bleed stopped but after 24 hours patient rebleeds from a fresh point above the previously banded ulcer. (e) The new point of bleeding is caught inside the band. (f) Two bands are seen applied separately. (g) An inflowing perforator of the diameter of 3 mm is seen coming from the lateral wall of rectum before banding. (h) The diameter of rectal varices became smaller and more numerous as they were followed downwards the anorectal junction. At 4 cm distance the rectal varices are seen going towards the prostate.

**Figure 5:**
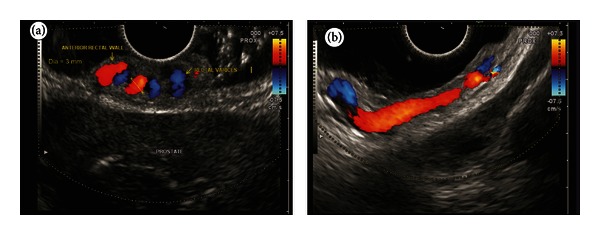
(a) Submucosal varices noted in a person who has undergone surgery for hemorrhoids. Presence of fresh blood was noted but endoscopy showed no rectal varices. (b) A long submucosal course of rectal varices is seen coming from the lateral wall of the rectum towards the anterior wall.

In three cases detection was possible by endoscopy. EUS helped in identifying RVs in two. The clinical, endoscopic and EUS features of the patients are given in Table 1. Hemodynamic evaluation showed four areas of rectal venous circulation: inflow area (from 10 to 8 cm), downflow area (from 8 to 6 cm), outflow area in the lower rectum (6 to 4 cm), and outflow area in the anal canal. The EUS appearance in inflow area corresponded with highest point of RVs on endoscopy and in downflow area corresponded with endoscopic presence of multiple submucosal RVs (Figures 2(a), 2(b), and 2(c)). The EUS appearance in the outflow area in lower rectum corresponded with numerous smaller submucosal RVs and perforators, and the EUS findings in anal canal corresponded with small submucosal vessels and outflowing perforators through the middle part of anal canal (Figures 3(a) and 3(b)). The first three cases presented with LGIB for the first time and multiple EVL was done. The fourth case presented with recurrent LGIB endoscopic appearance suggested Dieulafoy ulcer and EUS confirmed presence of RVs. His bleeding stopped after banding but he had rebled after 48 hrs from a similar spot higher up in rectum, which was also banded (Figures 4(a)–4(h)). The last case presented with persistent LGIB with presence of fresh blood in rectum after surgery of hemorrhoids. Two bands were applied in anterior wall of rectum after the detection of varices by EUS (Figures 5(a) and 5(b)). None of the five cases had recurrence in a 6-month followup. 

In this series 5 cases of RVs were detected (endoscopic detection *N* = 3, EUS detection *N* = 2). The detection of endoscopically inevident RVs was possible only by EUS in two cases, and potentially hazardous application of endoclips or coagulation methods on a bleeding point was avoided [1, 6, 7, 12]. The application of band on a normal looking mucosa on endoscopically inevident rectal varices stopped bleeding in a case of LGIB operated for internal hemorrhoids [8]. In this series the EUS was able to demonstrate the similarity of rectal venous circulation to esophageal venous circulation (Figures 1(a) and 1(b)) [13, 14]. The inflow area showed inflowing perforators communicating the pararectal varices with submucosal RVs and the downflow area showed the presence of RVs till the anorectal junction. The outflow area in lower rectum showed outflowing perforators in anterior and lateral wall of rectum, and the outflow area in anal canal demonstrated outflowing perforators.

**Figure 6:**
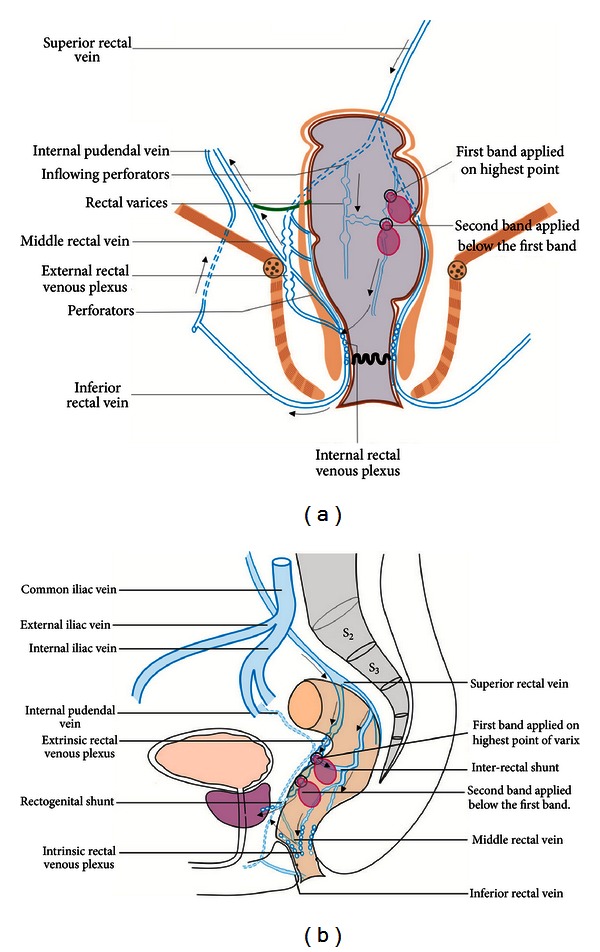
The direction of flow in rectal varices as shown in the figure is generally hepatofugal. The rectal varices are formed from these upper submucosal veins of intrinsic rectal venous plexus. From both the plexuses the portal hemorrhoidal blood works into systemic circulation through two portosystemic shunts (recto genital and inter rectal). The recto genital communication connect the rectal venous plexus with vesicoprostatic or vaginal venous plexus. The inter rectal communications occur between the three rectal veins. In rectal varices the banding should be done from above downwards.

No standard algorithm is suggested for management of RVs. Balloon-occluded retrograde transvenous obliteration is aimed at obliterating the feeder vessel of superior rectal vein draining into inferior mesenteric vein while endoscopic obliteration takes care of submucosal blood vessels [10]. The hemodynamic evaluation can offer therapeutic advantage before the selection of endoscopic or interventional radiological therapy [13]. In this series hemodynamic evaluation helped in selection of banding closer to the feeder vessel near the inflow area at the highest point. The site, size, and direction of flow of RVs were evaluated, but the confirmation of inflow around 10 cm distance from anus was sufficient for selection of therapy, and banding of the highest point of RVs was done. This approach is contrary to the approach in a retrospective study where no hemodynamic evaluation was done and banding of the RVs was done close to the lowest point at anorectal junction [15]. This approach adopted in our series is analogous to obliteration of esophageal varices from the lowest point where the blood flow is from below upwards (Figures 6(a) and 6(b)). 

## 4. Conclusion

To conclude EUS is helpful in identifying EIERV and in HDE of RVs. The identification of inflowing perforator to RVs at about 10 cm distance in the rectum is helpful in selecting the optimum site of RVs banding. The banding of RVs should be done from above downwards. 

## Figures and Tables

**Table 1:** Baseline characteristics and endoscopic and EUS findings.

Headings	Case 1	Case 2	Case 3	Case 4	Case 5
Age/sex	35/M	40/F	50/M	38/F	23/M
Cause of portal hypertension	Cirrhosis (HCV)	Cirrhosis (HBV)	EHPVO, Cirrhosis (HBV)	EHPVO	Cirrhosis (alcohol)
Presenting symptom	LGIB 1st episode	LGIB 1st episode	LGIB 1st episode	Persistent LGIB after hemorrhoidectomy	Recurrent LGIB
Endoscopy finding	Tortuous varices	Tortuous varices	Tortuous varices	Normal rectal mucosa with presence of fresh blood	Appearance of Dieulafoy's ulcer

EUS findings					

(a) Inflow zone—size and number	3-4 (mm)/one	3-4 (mm)/one	2-3 (mm)/one	3-4 (mm)/one	3-4 (mm)/one
(b) Downflow zone—size and number	2-3/multiple	2-3/multiple	2-3/multiple	2-3/multiple	2-3/multiple
(c) Outflow zone (LR)—size and number	1-2/multiple	1-2/multiple	1-2/multiple	1-2/multiple	1-2/multiple
(d) Outflow zone—anal canal and number	Absent	Absent	~1 mm/multiple	Absent	~1 mm/multiple

HBV: hepatitis B virus, HCV: hepatitis C virus, inflow zone = 8–10 cm distance from anal verge, downflow zone = 6–8 cm distance from anal verge, outflow zone—upper rectum = 4–6 cm from anal verge, Outflow zone—anal canal = 1–4 cm from anal verge, LR: lower rectum.

## References

[1] Hosking SW, Johnson AG, Smart HL, Triger DR (1989). Anorectal varices, haemorrhoids, and portal hypertension. *The Lancet*.

[2] Chawla Y, Dilawari JB (1991). Anorectal varices—their frequency in cirrhotic and non-cirrhotic portal hypertension. *Gut*.

[3] Goenka MK, Kochhar R, Nagi B, Mehta SK (1991). Rectosigmoid varices and other mucosal changes in patients with portal hypertension. *American Journal of Gastroenterology*.

[4] Aigner F, Gruber H, Conrad F (2009). Revised morphology and hemodynamics of the anorectal vascular plexus: impact on the course of hemorrhoidal disease. *International Journal of Colorectal Disease*.

[5] McCormack TT, Bailey HR, Simms JM, Johnson AG (1984). Rectal varices are not piles. *British Journal of Surgery*.

[6] Vianna A, Hayes PC, Moscoso G (1987). Normal venous circulation of the gastroesophageal junction: a route to understanding varices. *Gastroenterology*.

[7] Shafik A, Mohi-el-Din M (1985). A new concept of the antomy of the anal sphincter mechanism and the physiology of defaecation. XXIV. Haemorrhoidal venous plexuses; anatomy and role in haemorrhoids. *Colorproctology*.

[8] Azar C, Khalifeh M, Al-Kutoubi MA, Sharara AI (2006). Recurrent massive haemorrhage from an endoscopically inevident isolated rectal varix. *Digestive and Liver Disease*.

[9] Sato T, Yamazaki K, Toyota J, Karino Y, Ohmura T, Akaike J (2007). Diagnosis of rectal varices via color doppler ultrasonography. *American Journal of Gastroenterology*.

[10] Sharma M, Somasundaram A (2010). Massive lower GI bleed from an endoscopically inevident rectal varices: diagnosis and management by EUS (with videos). *Gastrointestinal Endoscopy*.

[11] Dhiman RK, Saraswat VA, Choudhuri G, Sharma BC, Pandey R, Naik SR (1999). Endosonographic, endoscopic, and histologic evaluation of alterations in the rectal venous system in patients with portal hypertension. *Gastrointestinal Endoscopy*.

[12] Sato T, Yamazaki K, Toyota J, Karino Y, Ohmura T, Akaike J (2009). Evaluation of therapeutic effects on rectal varices using percutaneous color Doppler ultrasonography. *Hepatology Research*.

[13] Wiechowska-Kozłowska A, Białek A, Milkiewicz P (2009). Prevalence of “deep” rectal varices in patientswith cirrhosis: an EUS-based study. *Liver International*.

[14] Norton ID, Andrews JC, Kamath PS (1998). Management of ectopic varices. *Hepatology*.

[15] Levine CD, Gonzales RN, Wachsberg RH (1997). CT evaluation of pararectal varices. *Journal of Computer Assisted Tomography*.

